# Supplementary material for: Nonreciprocal field theory for decision-making in multi-agent control systems
Source: Nat Commun. 2025 Sep 26;16:8450. doi: 10.1038/s41467-025-63071-4 (PMC12474890; doi:10.1038/s41467-025-63071-4)
Supplement: Supplementary file 1 — Supplementary Information [file 41467_2025_63071_MOESM1_ESM.pdf]

Supplementary Information
"Nonreciprocal field theory for decision-making in multi-agent control systems"

Andrea Lama*
Modeling and Engineering Risk and Complexity, Scuola Superiore Meridionale, Naples, 80125, Italy

Mario di Bernardo†
Department of Electrical Engineering and ICT, University of Naples Federico II, Naples, 80125, Italy

Sabine. H. L. Klapp‡
Institute for Physics and Astronomy, Technische Universität Berlin, 10623 Berlin, Germany
(Dated: September 1, 2025)

In this Supplementary Information we provide, first, background information and additional results for our agent-based model. Second, we discuss several aspects of the continuum model regarding, in particular, the derivation and the linear stability analysis. Third, we discuss relations between continuum and agent-based results used in the context of the more general design rules considered in Fig. 5 of the main text. Finally, we present a list of the supplementary videos. A list of references list is given at the end of this Supplemental Information.

CONTENTS

I. Agent-based model 2
   A. Comparison with other models 2
   B. Guidelines for parameter choices 2
   C. Impact of the number of herders 2
   D. Impact of the geometry of the goal region 3
   E. Robustness of results for the fraction of confined targets 4

II. Continuum theory 5
   A. Derivation of coupling functions 5
      1. Higher order terms in the gradient expansion 7
   B. Analysis in the absence of decision-making 8
   C. Sharpness of the herder-target interface 9

III. Pattern design beyond simple containment 11
   A. Continuum: choices for $v_1(x)$ 12
   B. Agent dynamics: decison-making rules 13
   C. Linear stability analysis for the case of constant coefficients $\tilde{v}_1$ and $\tilde{v}_2$ 14

IV. List of Supplementary videos 17

References 17

* andrea.lama-ssm@unina.it
† mario.dibernardo@unina.it
‡ sabine.klapp@tu-berlin.de

I. AGENT-BASED MODEL

A. Comparison with other models

In this section we discuss our agent-based model (see Methods) in the light of earlier models describing shepherding dynamics, particularly the one considered by some of the authors in [1]. The latter model, along with other established ones [2–4], includes elements that are plausible on the microscopic level but make the derivation of a continuum theory unnecessarily complicated. It is for this reason that we have chosen here to simplify the model. The specific modifications compared to [1] are as follows:

First, and most importantly, we have removed a cooperation strategy between herders that prevents two or more of them to chase the same target. We recall [see Eq. (1) of the main text] that a herder i at position $\mathbf{H}_i$ only considers targets a at positions $\mathbf{T}_a$ within its sensing radius ξ (i.e., $|\mathbf{H}_i - \mathbf{T}_a| \leq \xi$). Ref. [1] additionally involves a proximity-based rule: even if another target b at $\mathbf{T}_b$ is within herder i's sensing region, it will not be followed by the herder i if there is another herder j (inside herder i's sensing region) that is closer to b (i.e., if $|\mathbf{H}_j - \mathbf{T}_b| < |\mathbf{H}_i - \mathbf{T}_b|$). This mechanism promotes the desired pattern, namely a spreading of herders around the circular arrangement of targets, but is hard to translate to the continuum level. Upon removing this cooperative element, multiple herders can cluster around the same target that locally maximizes its distance from the origin. To qualitatively reproduce the herder spreading behavior, we introduce noise into the herders' dynamics – an element often absent in earlier works [1–4]. For simplicity, we apply the same noise term used for target dynamics (see Eqs. (7-8) in the Methods). This noise mitigates the tendency of herders to cluster while chasing the same target; this partially recreates the desired spreading behavior. At the same time, the noise can be easily translated to the continuum by a diffusive term. We note that the introduction of noise within herder dynamics is not uncommon; it is also used, e.g., to model environmental disturbances in collective animal behavior [5, 6] or measurement errors in swarm robotics [7, 8].

As a second modification compared to [1], we have eliminated the saturation of the herder speed, $\dot{\mathbf{H}}_i$, that was implemented in [1] to prevent unrealistically high values when varying the herders' sensing radius ξ over more than an order of magnitude. In the present work, ξ was fixed to a moderate value, making the saturation redundant.

Finally, we here employ a finite simulation box with periodic boundary conditions, consistent with the large body of literature in active and nonreciprocal systems (see, e.g., [9]). Given the presence of a "special" point being the center of the goal region, the resulting system can be visualized as a torus with one special point (i.e., the origin).

B. Guidelines for parameter choices

Equations (7-8) of the Methods contain multiple parameters that we have fixed based on the following considerations.

The domain size L and number of targets N_T were chosen to ensure the average target density $(N_\mathrm{T})/L^2$ remains sufficiently high to prevent issues arising from herders' limited sensing capabilities. This is achieved by maintaining N_T above the critical threshold $N_\mathrm{T}^\mathrm{low}(L,\xi)$ described in [1].

Following standard practices in the control and robotics literature [10], we set the herder sensing radius ξ larger than the target repulsion range λ (i.e., $\xi > \lambda$). Additionally, we ensure that target-herder nonreciprocal interactions dominate over noise effects by maintaining $k^T\lambda^2 \gg D$ and $k^H\xi^2 \gg D$. The soft, reciprocal, short-range repulsion is configured to dominate other interactions ($k^\mathrm{rep} \gg k^\mathrm{H}, k^\mathrm{T}$) while maintaining the hierarchy of ranges, $\sigma < \lambda < \xi$. The concrete values for these parameters are given in the Methods.

C. Impact of the number of herders

The numerical results in the main text have been obtained by choosing equal numbers of targets and herders, that is, $N_\mathrm{T} = N_\mathrm{H} = 400$. Here we present results for systems where $N_\mathrm{H} < N_\mathbf{T}$, which is the common choice in the control literature on shepherding [1, 4, 11]. Figure S1 shows representative long-time configurations for different values of the control parameters γ and δ and $N_\mathrm{H} = 100$ (panel (a)-(d)) and $N_\mathrm{H} = 200$ (panel (e-h)), while $N_\mathrm{T} = 400$ is fixed. Visual inspection of the respective snapshots suggests that the qualitative features of the dynamics observed for $N_\mathrm{H} = 400$ (see Fig. 3 of the main text) remain. This robustness is further confirmed by the radial density profiles $\hat{\rho}^\mathrm{T}(r)$ and $\hat{\rho}^\mathrm{H}(r)$ shown in the insets, which exhibit spatial structures similar to those observed in our main analysis.

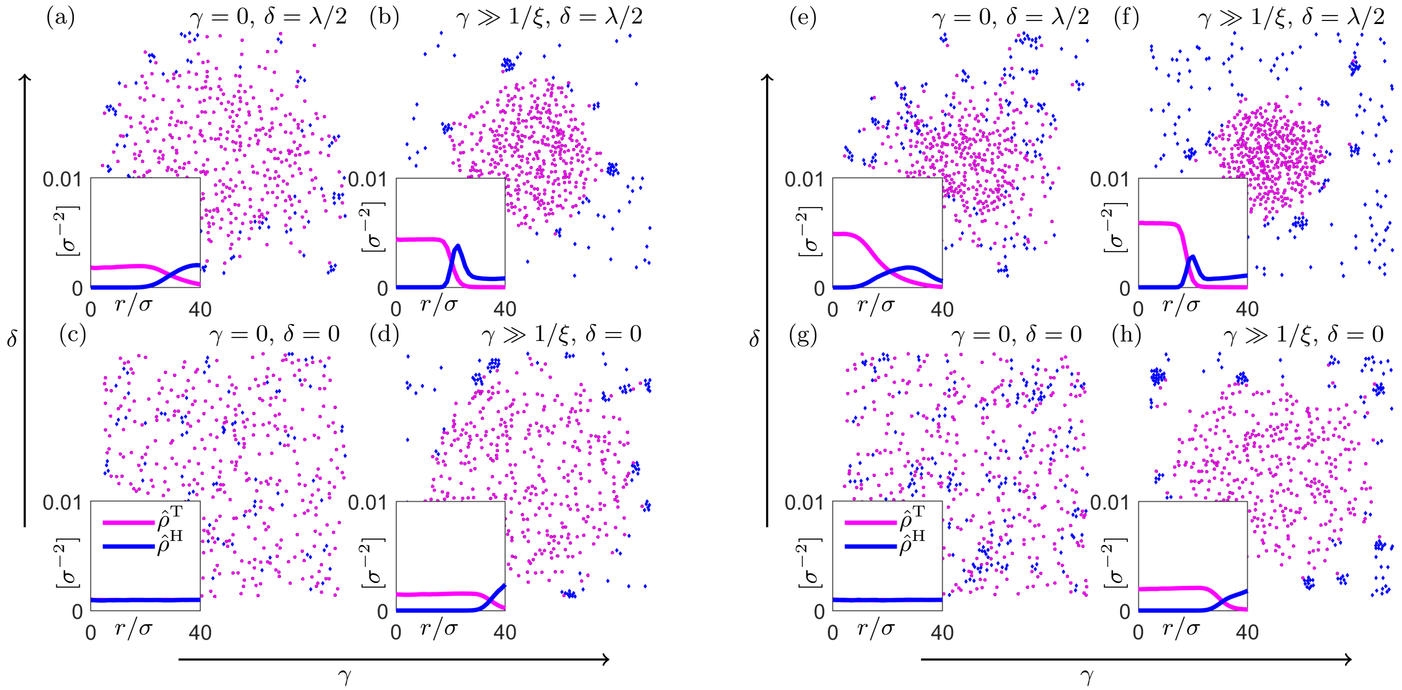

FIG. S1: Snapshots of typical long time configurations for representative values of the control parameters γ and δ for $N_{\mathrm{H}} = 100$ (a-d) and $N_{\mathrm{H}} = 200$ (e-h); in both cases $N_{\mathrm{T}} = 400$ as for the main text. The insets show the time-averaged and angle-averaged densities of agents, $\hat{\rho}^{\mathrm{T}}(r)$ and $\hat{\rho}^{\mathrm{H}}(r)$, as a function of the distance r from the origin for $r \leq L/2$. The simulations show no significant difference with respect to those presented in the main text.

D. Impact of the geometry of the goal region

The agent-based results in Fig. 3 of the main text are based on the assumption of a circular goal region centered around the origin, consistent with the established shepherding literature [1, 4]. Importantly, this circular geometry influences both steps of the decision-making process of the herders, namely (i), the selection rule for $\mathbf{T}_i^*$ [Eq. (1) of the main text] that is based on the distance of targets from the origin, and (ii), the specification of the herder's positioning relative to $\mathbf{T}_i^*$ that is crucial for the trajectory planning.

To explore the possible role of geometry on the shepherding dynamics we here present, as an alternative, agent-based results for a rectangular geometry. Concretely, the simulations are still performed in two dimensions (in a square box of side length L with periodic boundary conditions), but the goal region is now defined as a stripe centered around the origin at $x = y = 0$ and extending along the y-direction. To realize this goal, each herder i now selects its target $\mathbf{T}_i^*$ based on the maximum distance of each target a from the y-axis, that is, based on the x-component $\mathbf{T}_{a,x}$. The selection rule then becomes

$$\mathbf{T}_i^* = \frac{\sum_{a \in N_{i,\xi}} e^{\gamma(|\mathbf{T}_{a,x}| - |\mathbf{H}_{i,x}|)} \mathbf{T}_a}{\sum_{a \in N_{i,\xi}} e^{\gamma(|\mathbf{T}_{a,x}| - |\mathbf{H}_{i,x}|)}}. \tag{S1}$$

Furthermore, herders position themselves behind $\mathbf{T}_i^*$ with shift vector $\widehat{\mathbf{T}}_i^* = \mathrm{sign}(\mathbf{T}_{i,x}^*)\hat{\mathbf{x}}$, where $\hat{\mathbf{x}}$ is the unit vector in x-direction. Accordingly, the control input $\mathbf{u}_i$ for each herder becomes

$$\mathbf{u}_i = -\left(\mathbf{H}_i - (\mathbf{T}_i^* + \delta\, \mathrm{sign}(\mathbf{T}_{i,x}^*)\hat{\mathbf{x}})\right) \tag{S2}$$

The remaining dynamical elements from Eq. (7-8) in the Methods and discussed in the main text are preserved. Figure S2 presents snapshots of the agent-based simulations for different values of control parameters γ and δ, along with the corresponding agent densities along the x direction, $\hat{\rho}^{\mathrm{T}}(x)$ and $\hat{\rho}^{\mathrm{H}}(x)$. The numerical density calculations follow a methodology similar to that used for the radial densities $\hat{\rho}^{\mathrm{T}}(r)$ and $\hat{\rho}^{\mathrm{H}}(r)$ in the circular case, with two key differences: first, we compute histograms using Cartesian coordinates (x, y) rather than polar coordinates (r, θ), and second, we integrate over the y axis instead of averaging over the polar angle θ. Additionally, unlike the radial case

where geometric constraints limited our analysis to agents within a circle of radius $L/2$, here we include all agents in our density calculations. The density profiles exhibit striking similarities between the circular and rectangular geometries. Notably, the rectangular configuration's snapshots demonstrate that the system can be effectively reduced to a one-dimensional setting through y-axis averaging. This simplification establishes direct connections to our one-dimensional continuum framework and supports our assertion that one-dimensional field theories can capture the essential features of the shepherding problem.

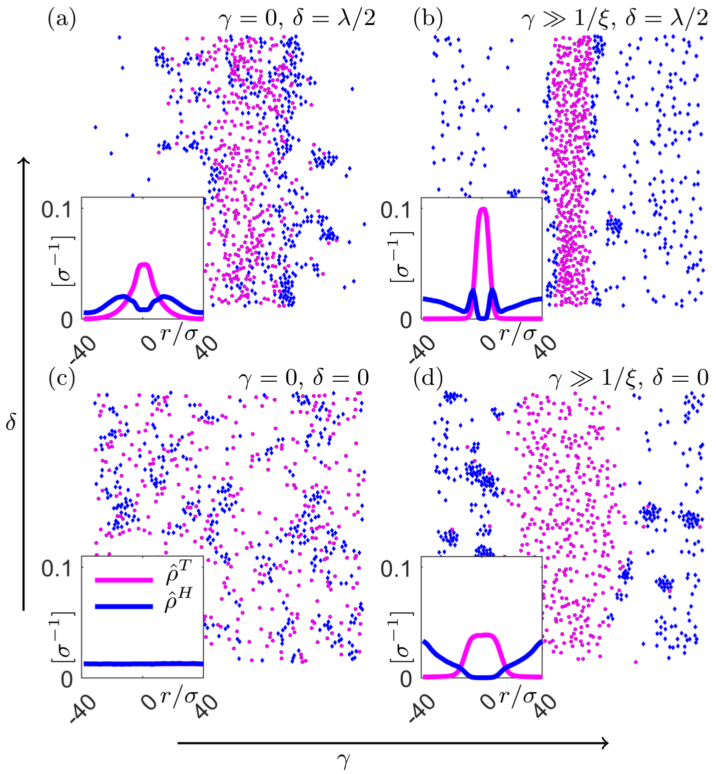

FIG. S2: Long-time configurations of the agent-based system for exemplary values of the two control parameters γ and δ; the insets represent the time-averaged histograms of targets $\hat{\rho}^T(x)$ and herders $\hat{\rho}^H(x)$ as a function of the relative position from the y-axis x, obtained from the 2 dimensional histogram after integrating over the y direction. At $\gamma = \delta = 0$ we observe a disordered state with homogeneous density distribution (c). The other panels show that, as soon as δ or γ are non zero, inhomogeneous configurations are reached (consistent with Eq. (6) of the main text), where we observe that herders tend to surround targets.

E. Robustness of results for the fraction of confined targets

A key order parameter to characterize the inhomogeneous steady state resulting from the shepherding process is the fraction of confined targets, $\chi(R)$, within a circle of radius R centered around the origin, see Fig. 4(a) of the main text. In these agent-based calculations, R has been chosen based on the fitted density profile of the targets in the case with the largest considered values for both γ and δ. Here we present data for two alternative choices of R. These are inspired by the fact that the targets in our model are represented by mutually repelling disks. It is well known that hard-disk systems, in thermal equilibrium, exhibit a first-order freezing transition from a fluid into a solid state [12]. Considering the target distribution within the goal region as solid-like, we can deduce the radii related to the minimum and maximum area fraction in such a solid, yielding $R^{\min} \sim \sqrt{N_\mathrm{T}\sigma^2/(1.15\pi)}$ and $R^{\max} \sim \sqrt{N_\mathrm{T}\sigma^2/(0.9\pi)}$. The resulting phase diagrams (obtained after time - and ensemble averaging in the steady state) are shown in Fig. S3(a-b). Comparing with the original result we conclude that the precise choice of R has no significant impact on the structure of the diagram.

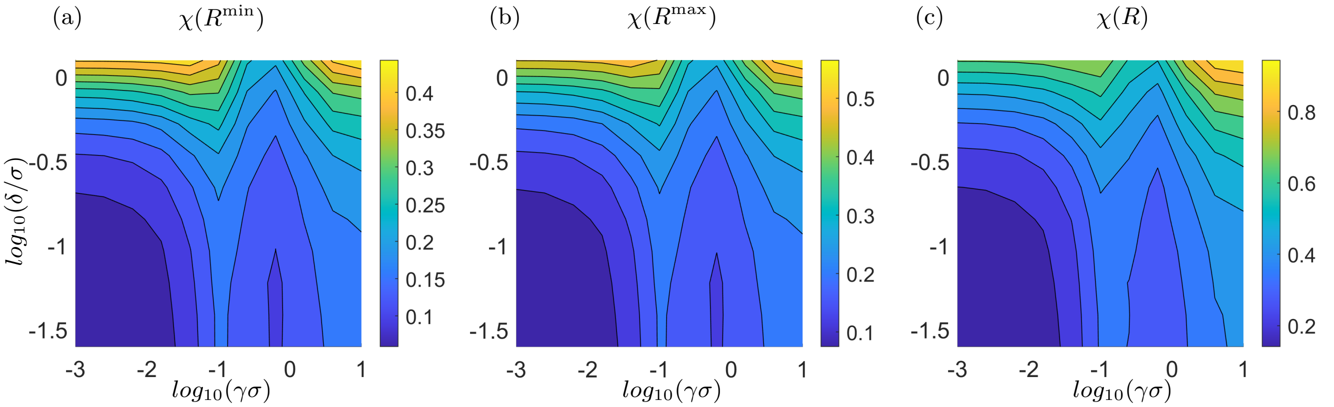

FIG. S3: Fraction of confined targets $\chi(R)$ for different choices of the size of the goal region, R, in the steady state of the agent-based simulations. (a-b) The values of R are derived from the minimum and maximum allowed densities for a solid phase of hard spheres in 2D [12]. These two values are $R^{\min} \sim \sqrt{N_{\mathrm{T}}\sigma^2/(1.15\pi)}$ and $R^{\max} \sim \sqrt{N_{\mathrm{T}}\sigma^2/(0.9\pi)}$. (c) Results for the value of R considered in the main text, which are the same of those shown in Fig. 4(a) of the main text. We can see that there is no significant difference in the behaviour of $\chi(\gamma, \delta)$.

II. CONTINUUM THEORY

A. Derivation of coupling functions

In this section we provide details on the derivation of the one-dimensional field equations (3-4) of the main text for the targets and herder densities, $\rho^{\mathrm{T}}(x,t)$ and $\rho^{\mathrm{H}}(x,t)$, respectively (see the Methods for a summary). The field equations for these conserved fields take the general form [9]

$$\partial_t \rho(x,t) = -\nabla\left[\langle F\rangle(x,t)\rho(x,t)\right] + D\nabla^2\rho(x,t) \tag{S3}$$

where the average force $\langle F\rangle(x,t)$ at position x and time t is derived from the microscopic forces $F_{i(a)}$ acting on individual agents through a mean-field assumption. Specifically, we employ the following "recipe":

$$F_i(t) = \sum_{j\in\mathcal{N}_i} F_{ji}(t) \rightarrow \langle F(x,t)\rangle = \int_{\mathbb{B}(x)} F(x,y)\rho(y,t)\,dy \tag{S4}$$

where $\mathbb{B}(x)$ denotes the finite interaction range between the agents centered around x. As explained in the Methods, the function $F(x,y)$ is translationally invariant (i.e., $F(x,y) = F(x-y)$ for the short-range and long-range repulsion in Eqs. (7-8) while it is not for the "force" imposed by the feedback control input in the dynamics of the herders. In any case, direct computation of the integral over y is not possible since the density $\rho(y,t)$ is not known a priori. Following standard procedures [9], we employ in all cases a gradient expansion

$$\rho(y) \simeq \rho(x) + \nabla\rho(x)(y-x) + \mathcal{O}(\nabla^2\rho(x)) \tag{S5}$$

where we take into account (at most) the term linear in the gradient.

We first apply the procedure of Eq. (S4) with Eq. (S5) to the pairwise repulsive forces $\mathbf{F}^{\mathrm{rep}}_{\mathrm{SR(LR)}}$ that share the same functional form [see Eq (9)] and, crucially, depend only on agents relative distances. Therefore, the corresponding kernels satisfy the condition of translational invariance; $F(x,y) = F(x-y)$. We also note that, for each x, we integrate over a domain in y that is symmetric around x, and that $F(x-y)$ is, in our case, an odd function in this domain. As an important consequence we find that the zeroth-order term in the gradient expansion of Eq. (S5) cancels out. Therefore, the leading non-zero contribution comes from the gradient term, yielding

$$\langle F^{\mathrm{rep}}_{\mathrm{SR(LR)}}\rangle(x) = \int_{x-\sigma}^{x+\sigma} F^{\mathrm{rep}}_{\mathrm{SR(LR)}}(x-y)(y-x)\,dy\,\nabla\rho(x) \tag{S6}$$

where we have used that $\nabla\rho(x)$ is independent of y and can therefore be pulled out of the integral.

From Eq. (S6) we obtain for the short-range (SR) average forces

$$k^{\mathrm{rep}}\langle F^{\mathrm{SR}}\rangle(x) = k^{\mathrm{rep}}\int_{x-\sigma}^{x+\sigma}\mathrm{sign}(x-y)(\sigma-|x-y|)(y-x)\nabla\rho(x)\,dy = -\alpha^{\mathrm{SR}}\nabla\rho(x) = -k^{\mathrm{rep}}\frac{\sigma^3}{3}\nabla\rho(x) \tag{S7}$$

Analogously, the contribution from the LR forces follows as

$$k^{\mathrm{T}}\langle F^{\mathrm{LR}}\rangle(x) = k^{\mathrm{T}}\int_{x-\lambda}^{x+\lambda}F^{\mathrm{rep}}_{\mathrm{LR}}(x-y)(y-x)\nabla\rho(x)\,dy = -\alpha^{\mathrm{LR}}\nabla\rho(x) = -k^{\mathrm{T}}\frac{\lambda^3}{3}\nabla\rho(x) \tag{S8}$$

We now turn to the feedback control term that represents decision-making. As discussed in the Methods, the corresponding kernel describing how the herders select the target to chase and how to place themselves relative to the target is not translationally invariant (since the decision-making depends on agent positions relative to the goal region at $x=0$), that is, $F(x,y)$ also depends separately on x and y. To simplify the calculations, We first take the control input from Eq. (2) of the main text in its 1D form, $u_i = -k^{\mathrm{H}}(H_i - T_i^* - \delta\mathrm{sign}(T_i^*))$, and invert the order of the $\mathrm{sign}(\cdot)$ and the weighted average relative to herder i, denoted as $(\cdot)_i^*$. This yields $u_i = -k^{\mathrm{H}}(H_i - T_i^* - \delta(\mathrm{sign}(T))_i^*)$. Notice that these two expressions are equivalent throughout most of the domain, specifically for $\xi \le |H_i| \le L/2-\xi$, with $L \gg \xi$. We then linearize the exponent and neglect the denominator in the selection rule (see Methods), yielding

$$F^{\mathrm{DM}}(x,y) = -k^{\mathrm{H}}(1+\gamma(|y|-|x|))(x-(y+\mathrm{sign}(y)\delta))\,. \tag{S9}$$

As a consequence of the lacking translational invariance, the symmetry arguments used above, which lead to the vanishing of the zeroth order term in the gradient expansion, do not apply any more. Thus, the general form of this term is given by

$$\langle F^{\mathrm{DM}}\rangle(x) = \int_{x-\xi}^{x+\xi}F^{\mathrm{DM}}(x,y)\left(\rho(x)+\nabla\rho(x)(y-x)\right)\,dy = g_1(x)\rho(x)+\nabla\rho(x)g_2(x) \tag{S10}$$

where $g_1(x)$ and $g_2(x)$ are functions depending explicitly on x (rather than constants as in the case of translational invariance discussed before). Clearly, $g_{1,2}(x)$ also depend on the parameters γ and δ, but we drop this here for notational ease.

To proceed, we differentiate the domain into three regions: (i) $x\in[-\xi,0]$ and $x\in[0,\xi]$, (ii) $x\in(\xi,L/2-\xi]$ and $x\in[-L/2+\xi,-\xi)$, (iii) $x\in(L/2-\xi,L/2)$ and $x\in[-L/2,-L/2+\xi)$. We here focus on the intervals $x\in[0,\xi]$ and $x\in(\xi,L/2-\xi]$, the other cases follow analogously. Starting with $g_1(x)$, we find for $0\le x\le\xi$

$$\begin{aligned}
g_1(x)|_{x\in[0,\xi]} =& -k^{\mathrm{H}}\int_{x-\xi}^{x+\xi}(1+\gamma(|y|-|x|))(x-(y+\mathrm{sign}(y)\delta))\,dy = \\
=& -k^{\mathrm{H}}\left(\int_{x-\xi}^{0}(1+\gamma(-y-x))(x-(y-\delta))\,dy + \int_{0}^{x+\xi}(1+\gamma(y-x))(x-(y+\delta))\,dy\right) = \\
=& k^{\mathrm{H}}\left(\delta(1-2\gamma x)(x-\xi)+\delta(x+\xi)+\gamma x(\xi^2-x^2)+\frac{2}{3}\gamma x^3\right)
\end{aligned} \tag{S11}$$

For the interval $\xi < x < L/2-\xi$, the calculations are simpler since y keeps the same sign throughout the integration domain. We obtain:

$$\begin{aligned}
g_1(x)|_{x\in(\xi,L/2-\xi]} =& -k^{\mathrm{H}}\int_{x-\xi}^{x+\xi}(1+\gamma(|y|-|x|))(x-(y+\mathrm{sign}(y)\delta))\,dy = \\
=& -k^{\mathrm{H}}\int_{x-\xi}^{x+\xi}(1+\gamma(y-x))(x-(y+\delta))\,dy = \\
=& k^{\mathrm{H}}\left(2\delta\xi+\frac{2}{3}\gamma\xi^3\right)
\end{aligned} \tag{S12}$$

Similar calculations can be performed for $g_2(x)$. Starting with the interval $0\le x\le\xi$, we find

$$
\begin{aligned}
g_2(x)|_{x\in[0,\xi]} =& - k^{\rm H}\int_{x-\xi}^{x+\xi}(1+\gamma(|y|-|x|))(x-(y+\text{sign}(y)\delta))(y-x)\,dy = \\
=& - k^{\rm H}\left(\int_{x-\xi}^{0}(1+\gamma(-y-x))(x-(y-\delta))(y-x)\,dy + \int_{0}^{\xi}(1+\gamma(y-x))(x-(y+\delta))(y-x)\,dy\right) = \\
=& k^{\rm H}\left(\delta(1-\gamma x)(\xi^2-x^2) + \frac{\delta\gamma+1}{3}2\xi^3 - \frac{2\gamma x}{3}(\xi^3-x^3) + \frac{\gamma}{2}(\xi^4-x^4)\right) \qquad \text{(S13)}
\end{aligned}
$$

whereas for $\xi < x < L/2-\xi$,

$$
\begin{aligned}
g_2(x)|_{x\in(\xi,L/2-\xi]} =& - k^{\rm H}\int_{x-\xi}^{x+\xi}(1+\gamma(|y|-|x|))(x-(y+\text{sign}(y)\delta))(y-x)\,dy = \\
=& - k^{\rm H}\int_{x-\xi}^{z+\xi}(1+\gamma(y-x))(x-(y+\delta))(y-x)\,dy = \\
=& k^{\rm H}\frac{2}{3}(\gamma\delta+1)\xi^3 \qquad \text{(S14)}
\end{aligned}
$$

Using the same same steps for the other intervals we finally obtain the expressions of $g_1(x)$ and $g_2(x)$ reported in Eqs. (15-16) in the Methods. As explained there, the functions $g_1(x)$ and $g_2(x)$ coincide with the functions $v_1(x)$ and $v_2(x)$ in Eq. (3-4) of the main text up to a constant factor derived from the adimensionalization of the equations; furthermore, to obtain $v_2(x)$ we have to subtract from $g_2(x)$ the (constant) contribution arising from the SR repulsion between herders and targets $\alpha^{\rm SR}$.

1. *Higher order terms in the gradient expansion*

In this section we briefly consider consequences of including higher order terms in the gradient expansion given in Eq. (S5). The field equations describing the dynamics of $\rho^{\rm H}(x,t)$ and of $\rho^{\rm T}(x,t)$ will then have the form

$$
\begin{aligned}
\partial_t\rho^{\rm T} =& \nabla\cdot\left[D^{\rm T}(\rho^{\rm T})\nabla\rho^{\rm T} + \tilde{k}^{\rm T}\rho^{\rm T}\nabla\rho^{\rm H} + \rho^{\rm T}\sum_{i=3}^{Z}w_i\nabla^{(i-1)}\rho^{\rm H}\right] \qquad \text{(S15)} \\
\partial_t\rho^{\rm H} =& \nabla\cdot\left[D^{\rm H}(\rho^{\rm H})\nabla\rho^{\rm H} - v_1(x)\rho^{\rm H}\rho^{\rm T} - v_2(x)\rho^{\rm H}\nabla\rho^{\rm T} + \rho^{\rm H}\sum_{i=3}^{Z}v_i(x)\nabla^{(i-1)}\rho^{\rm T}\right] \qquad \text{(S16)}
\end{aligned}
$$

where Z is maximum order of the expansion we consider, and w_i and $v_i(x)$ are the corresponding couplings. Notice that, as argued before, the coupling functions w_i have to be constant since all kernels related to the interactions in the target's dynamics are translationally invariant. On the other hand, the decision-making interaction kernel in the herders' dynamics is not translationally invariant; this yields the x-dependence of $v_i(x)$.

As shown in the main text, for the continuum equation system up to linear order in the gradients, there is no homogeneous steady state. Interestingly, this statement holds for *any* order Z of the gradient expansion. To see this, we evaluate Eqs. (S15) and (S15) at $\rho^{\rm H} = \rho_0^{\rm H}$, $\rho^{\rm T} = \rho_0^{\rm T}$, yielding

$$
\begin{aligned}
&\rho_0^{\rm T}\sum_{i=3}^{Z}w_i\nabla^{(i-1)}\rho_0^{\rm H} = 0 \qquad \text{(S17)} \\
&\rho_0^{\rm H}\sum_{i=3}^{Z}v_i(x)\nabla^{(i-1)}\rho_0^{\rm T} = 0, \quad \forall Z\geq 3 \qquad \text{(S18)}
\end{aligned}
$$

The above relations imply that Eq. (6) of the main text holds true at any order Z of the gradient expansion. We conclude that the "shepherding" inhomogeneities that we observe are *not* just a by-product of the order of truncation of the gradient expansion of Eq. S5; rather they result from the decision-making itself.

B. Analysis in the absence of decision-making

Without decision-making (i.e., $\gamma = 0$, $\delta = 0$), the interaction kernel Eq. (S9) becomes translationally invariant, that is,

$$F^{\mathrm{DM}}(x,y)|_{\gamma=0,\,\delta=0} = -k^{\mathrm{H}}(x-y). \tag{S19}$$

As a result, the functions $g_1(x)$ and $g_2(x)$ appearing in Eq. (S10) reduce to constants, and we recover expressions similar to those found for the repulsive interaction kernel. Specifically,

$$\begin{aligned} g_1(x) &= 0 \\ g_2(x) &= \frac{2}{3}k^{\mathrm{H}}\xi^3 = g_2^0 \end{aligned} \tag{S20}$$

The resulting equations can be written as

$$\partial_t \rho^{\mathrm{T}} = \nabla \cdot \left[D^{\mathrm{T}}(\rho^{\mathrm{T}}) \nabla \rho^{\mathrm{T}} + \tilde{k}^{\mathrm{T}} \rho^{\mathrm{T}} \nabla \rho^{\mathrm{H}} \right] \tag{S21}$$

$$\partial_t \rho^{\mathrm{H}} = \nabla \cdot \left[D^{\mathrm{H}}(\rho^{\mathrm{H}}) \nabla \rho^{\mathrm{H}} - v_2^0 \rho^{\mathrm{H}} \nabla \rho^{\mathrm{T}} \right] \tag{S22}$$

where the density-dependent couplings $D^{\mathrm{T}}(\rho^{\mathrm{T}})$ (with A=H,T) and the constants $\tilde{k}^{\mathrm{T}}$ and v_2^0 are defined in the Methods. The above equations involve nonreciprocal cross-diffusive couplings $\nabla \cdot [\rho^{\mathrm{A}} \nabla \rho^{\mathrm{B}}]$ akin of the nonreciprocal Cahn-Hillard model [9, 13]. The new coupling $\nabla \cdot [v_1(x) \rho^{\mathrm{T}} \rho^{\mathrm{H}}]$ arising from herders' decision-making is absent, as expected at $\gamma = \delta = 0$.

We now perform a linear stability analysis (LSA) around the homogeneous, stationary state $\rho^{\mathrm{H}}(x,t) = \rho_0^{\mathrm{H}}$ and $\rho^{\mathrm{T}}(x,t) = \rho_0^{\mathrm{T}}$ which corresponds to the trivial solution of Eqs. (S21) and (S22). To this end we introduce small fluctuations, i.e.,

$$\rho^{\mathrm{A}}(x,t) = \rho_0^{\mathrm{A}} + \delta\rho^{\mathrm{A}}(x,t) \tag{S23}$$

and linearize the field equations in these fluctuations, yielding

$$\partial_t \delta\rho^{\mathrm{T}}(x,t) = D^{\mathrm{T}}(\rho_0^{\mathrm{T}}) \nabla^2 \delta\rho^{\mathrm{T}}(x,t) + \rho_0^{\mathrm{T}} \tilde{k}^{\mathrm{T}} \nabla^2 \delta\rho^{\mathrm{H}}(x,t) \tag{S24}$$

$$\partial_t \delta\rho^{\mathrm{H}}(x,t) = D^{\mathrm{H}}(\rho_0^{\mathrm{H}}) \nabla^2 \delta\rho^{\mathrm{H}}(x,t) - v_2^0 \rho_0^{\mathrm{H}} \nabla^2 \delta\rho^{\mathrm{T}}(x,t) \tag{S25}$$

We expand the fluctuations in a Fourier series, assuming that the growth rate $\Omega(q)$ is the same for both fields, that is

$$\delta\rho^{\mathrm{A}}(x,t) = \int dq\, e^{iqx+\Omega(q)t} \hat{\rho}^{\mathrm{A}}(q)) \tag{S26}$$

Stability of the homogeneous, stationary state implies that the real part of the growth rate $\Omega(q)$, $\mathrm{Re}(\Omega)$, is negative for all nonzero wavenumbers q (note that fluctuations at $q = 0$ cannot occur anyway due to particle number conservation). Inserting the ansatz (S26) into the linearized field equations (S25), (S24) we obtain the eigenvalue problem

$$-q^2 \begin{bmatrix} D^{\mathrm{T}}(\rho_0^{\mathrm{T}}) & \tilde{k}^{\mathrm{T}} \rho_0^{\mathrm{T}} \\ -v_2^0 \rho_0^{\mathrm{H}} & D^{\mathrm{H}}(\rho_0^{\mathrm{H}}) \end{bmatrix} \mathbf{v}(q) = \Omega(q) \mathbf{v}(q) \tag{S27}$$

where the eigenvalues $\Omega(q)$ corresponding to the eigenvectors $\mathbf{v}(q)$ are given by

$$\Omega(q) = -q^2 \frac{1}{2} \left(\Omega_0 \pm \sqrt{\Delta} \right) \tag{S28}$$

with

$$\begin{aligned} \Omega_0 &= D^{\mathrm{T}}(\rho_0^{\mathrm{T}}) + D^{\mathrm{H}}(\rho_0^{\mathrm{T}}) \\ \Delta &= \left(D^{\mathrm{T}}(\rho_0^{\mathrm{T}}) - D^{\mathrm{H}}(\rho_0^{\mathrm{T}}) \right)^2 - 4 v_2^0 \tilde{k}^{\mathrm{T}} \rho_0^{\mathrm{T}} \rho_0^{\mathrm{H}} \end{aligned} \tag{S29}$$

For parameter choices corresponding to our microscopic model, where all short-range interactions as well as the long-range herder-target interaction are repulsive, we have $D^{\mathrm{A}}(\rho_0^{\mathrm{A}}) > 0$ (for A = H,T) and $\tilde{k}^{\mathrm{T}} > 0$. Further, $v_2^0 > 0$ which, together with the negative sign of the corresponding term in Eq. (S22), reflects the attraction of herders to the center-of-mass target.

We first consider the case of equal number densities, $\rho_0^{\mathrm{H}} = \rho_0^{\mathrm{T}}$, considered in the main text. In this case, we have $\Delta < 0$ (for $v_2^0 > 0$, $\tilde{k}^{\mathrm{T}} > 0$) such that the eigenvalues become imaginary. However, since $\Omega_0 > 0$, $\mathrm{Re}(\Omega)$ is negative. Thus, the homogeneous steady state is stable. This result of the LSA conforms with the outcome of our numerical simulations as shown in Fig. 3(c) of the main text. Only by incorporating decision-making (that is, by introducing a coupling of the form $\nabla \cdot [v_1(x)\rho^{\mathrm{T}}\rho^{\mathrm{H}}]$ with $\gamma \neq 0$, $\delta \neq 0$) we achieve an inhomogeneous steady state characteristic of shepherding.

Similar considerations apply when we vary the densities ρ_0^{T}, ρ_0^{H} (keeping the total mass constant), or when we vary the constants v_2^0 and $\tilde{k}^{\mathrm{T}}$ (keeping them positive). In all of these cases, we find $\mathrm{Re}(\Omega) < 0$. An overview of the results obtained by numerical solution of Eq. (S28) is given in Fig. S4.

Still, it is interesting to investigate whether and at which coupling parameters the real part of the growth rate can become positive at all. Inspection of Eq. (S28) shows that this can only occur if Δ is positive and its square root exceeds the (positive) term Ω_0. A necessary requirement is that either $\tilde{k}^{\mathrm{T}} < 0$ (i.e, targets are attracted by herders) or $v_2^0 < 0$ (i.e, herders are repelled by targets), in contradiction to what we have assumed so far. The corresponding parameter regions are indicated in Fig. S4.

Finally, regarding the imaginary part of the growth rate, $\mathrm{Im}(\Omega)$, we see from Fig. S4 that there are no regions where both, $\mathrm{Re}(\Omega) > 0$ *and* $\mathrm{Im}(\Omega) \neq 0$, since the respective conditions contradict each other. Therefore, for the system without decision-making considered here, we do not find traveling patterns that have been observed in nonreciprocal field theories of phase separating systems [9, 13]. A particularly prominent example featuring traveling patterns is the non-reciprocal Cahn-Hilliard (NRCH) model [9, 13, 14], and indeed, our present model (S21)-(S22) without decision making has important similarities with the NRCH. As in the NRCH, the present model is mass-conserving and the cross-couplings ($\tilde{k}^{\mathrm{T}}$, $-v_2^0$) have different signs, yielding antagonistic (i.e., anti-reciprocal) couplings between different species.

However, there is a crucial difference with our model. In the NRCH, which is a generalized model of phase separation in binary mixtures, at least one of the intraspecies couplings is negative, reflecting attractive forces within agents of the same type. This leads to phase separation and the formation of a stable interface between the two phases (realized by a finite surface tension term in the NRCH) already for reciprocal cross-couplings. The underlying phase separation in the NRCH is a crucial prerequisite for the appearance of traveling patterns at high nonreciprocity [14].

In contrast, in our model (S21)-(S22), the prefactors related to intraspecies couplings are both positive due to the underlying repulsive (excluded-volume) interactions. This precludes the occurrence of traveling patterns (see Fig. 9 in [14]). We expect, however, that such patterns could occur when we add, e.g., adhesion between the targets. We will later show (see Sec. III of the SI) that traveling patterns can indeed occur for appropriate choices of the decision-making coupling term.

C. Sharpness of the herder-target interface

As shown in Fig. 4(d) of the main text, our continuum theory in presence of decision-making predicts the maximum sharpness of the target-herder interface, σ/Δ, at large values of γ and small values of δ. In contrast, at the agent-based level the maximum is found at large values of both, γ and δ. To explain this difference between the two levels of description we here present an argument based on the functions $v_1(x)$ and $v_2(x)$ entering the field equations Eqs. (3-4) of the main text in presence of decision-making. As we will see, these coupling functions have, to some extent, competing roles.

To this end, we consider the following simplified scenario. We assume that the targets have already arranged in a confined situation, that is, $\rho^{\mathrm{T}}(x)$ can be described by a hyperbolic-tangent profile, compatible with what is observed at long times in Fig. 2(d). Assuming this profile to be static (i.e., $\partial_t \rho^{\mathrm{T}} = 0$) we can focus on the dynamics of herders alone (once this is understood, we can infer the consequences for the sharpness of the interface).

As a further simplification of the herder dynamics, we approximate the function $v_1(x)$ by $\tilde{v}_1 \mathrm{sign}(x)$ and $v_2(x) = \tilde{v}_2$, as motivated by the actual shape of these functions shown in Fig. S5(a). The definition of the (positive) amplitudes $\tilde{v}_1$ and $\tilde{v}_2$ is also indicated in Fig. S5(a). Neglecting short-ranged contributions contained in $D^{\mathrm{H}}(\rho^{\mathrm{H}})$, the herder profile then evolves according to

$$\partial_t \rho^{\mathrm{H}} = \nabla \cdot [D' \nabla \rho^{\mathrm{H}} - \tilde{v}_1 \mathrm{sign}(x) \rho^{\mathrm{H}} \rho^{\mathrm{T}} - \tilde{v}_2 \rho^{\mathrm{H}} \nabla \rho^{\mathrm{T}}] \tag{S30}$$

where $D' = D\frac{\tau}{\sigma^2}$ is the adimensionalised diffusion coefficient. Focusing on the region $x \geq 0$, we find from Eq. (S30) that the term involving $\tilde{v}_1$ drives the herders "to the right" (i.e., larger values of x), whereas the term involving $\tilde{v}_2$

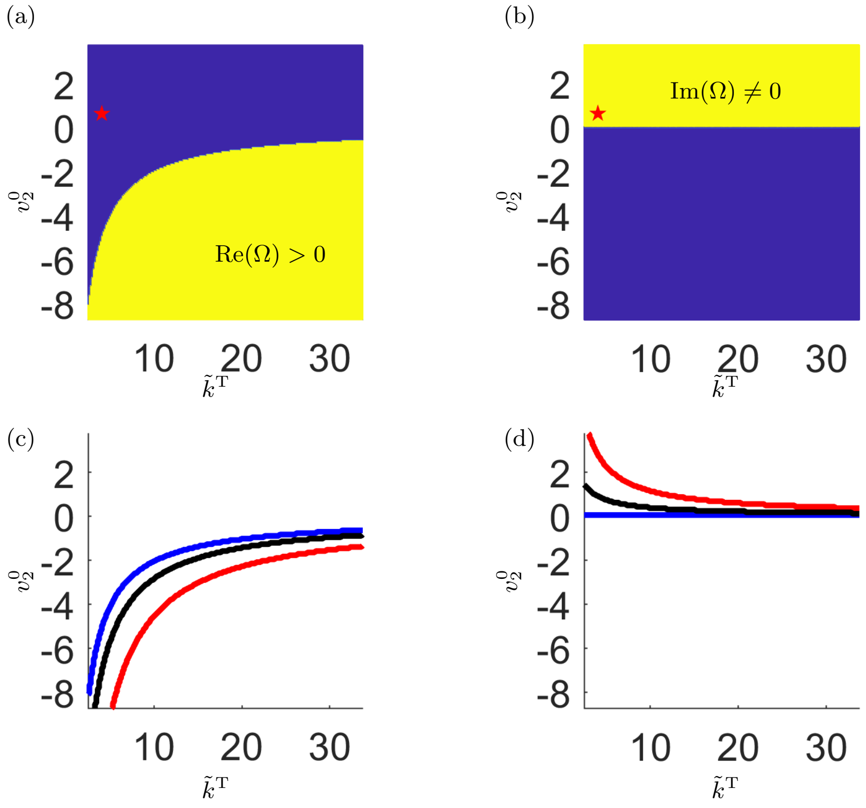

FIG. S4: Results of the LSA around the homogeneous, steady state ($\rho^{\mathrm{T}}(x) = \rho_0^{\mathrm{T}}$, $\rho^{\mathrm{H}}(x) = \rho_0^{\mathrm{H}}$) in the absence of decision-making (i.e., $\gamma = 0$, $\delta = 0$) in the parameter plane spanned by $\tilde{k}^{\mathrm{T}}$ and v_2^0. Parts (a) and (b) refer to the case $\rho_0^{\mathrm{T}} = \rho_0^{\mathrm{H}} = 0.5\rho_0$ (with $\rho_0 = 0.5/\sigma$) considered in the main text, with the red star indicating the corresponding parameter combination $(\tilde{k}^{\mathrm{T}}, v_2^0)$. Yellow regimes indicate where the real part of one of the growth rates is positive (a) or the imaginary part is nonzero (b). It is seen that the two regimes do not overlap. Parts (c) and (d) refer to the case of different densities (with the total density being the same as before). Here we show the curves dividing regimes with $\mathrm{Re}(\Omega) > 0$ from $\mathrm{Re}(\Omega) \leq 0$ (c) and $\mathrm{Im}(\Omega) \neq 0$ from $\mathrm{Im}(\Omega) = 0$) (d) for three cases: $\rho_0^{\mathrm{T}} = 0.1\rho_0$ (red), $\rho_0^{\mathrm{T}} = 0.5\rho_0$ (blue), and $\rho_0^{\mathrm{T}} = 0.8\rho_0$ (black), with $\rho_0^{\mathrm{H}} = \rho_0 - \rho_0^{\mathrm{T}}$. In all cases, the real part is negative as long as $v_2^0 > 0$, which coincides with the condition for a nonzero imaginary part.

drives the herders towards higher values of $\rho^{\mathrm{T}}(x)$, which means to move to the "left". In this sense, the two terms have conflicting roles.

If the term $\propto \tilde{v}_1$ dominates (i.e., $\tilde{v}_2 = 0$, or $\tilde{v}_1 \gg \tilde{v}_2$), the herders will all move to the right-most region and push the targets to the left. This will support the system in developing a sharp herder-target interface. However, if we increase $\tilde{v}_2$ from small values, the herders have to find a balance between the need to move right according to $\tilde{v}_1$, and to move left according to $\tilde{v}_2$. In this case, the herders will distribute themselves around the interface. Therefore, and since the herders exert a long-range repulsion on the targets, the interface will spread out, causing a decrease of the sharpness.

The preferred localization of herders depending on $\tilde{v}_1$ and $\tilde{v}_2$ can be seen more explicitly when we solve the one-dimensional Fokker-Planck Eq. (S30), yielding the steady-state solution [[15]]

$$\begin{aligned} \rho^{\mathrm{H}}(x) &\sim \exp\left[-V(x)/D'\right] \\ &\equiv \exp\left[\left(\tilde{v}_2\rho^{\mathrm{T}}(x) + \tilde{v}_1\int_0^x \rho^{\mathrm{T}}(x')\,dx'\right)/D'\right] \end{aligned} \tag{S31}$$

where $V(x)$ may be interpreted as an effective potential. The minima of $V(x)$ indicate where the herders tend to accumulate. In Fig. S5(c-d) we show that, by increasing $\tilde{v}_2$ relative to $\tilde{v}_1$, the minimum of $V(x)$ moves towards positions with higher values of $\rho^{\mathrm{T}}(x)$. Specifically, the minimum then occurs at the reflection point of the target profile, i.e., the center of the interface. Herders accumulating around this minimum disturb the distribution of targets

(through herder-target repulsion), which effectively leads to a "spreading" of the target-herder interface. We conclude that the ratio $\tilde{v}_1/\tilde{v}_2$ should be a good indicator of the sharpness, σ/Δ.

To close the argument we need to investigate how $\tilde{v}_1/\tilde{v}_2$ depends on γ and δ. The results are shown in Fig. S5(b). It is seen that the largest values of $\tilde{v}_1/\tilde{v}_2$ indeed are reached for large γ and small δ values. This is completely consistent with the corresponding dependencies of σ/Δ shown in Fig. 4(d) of the main text.

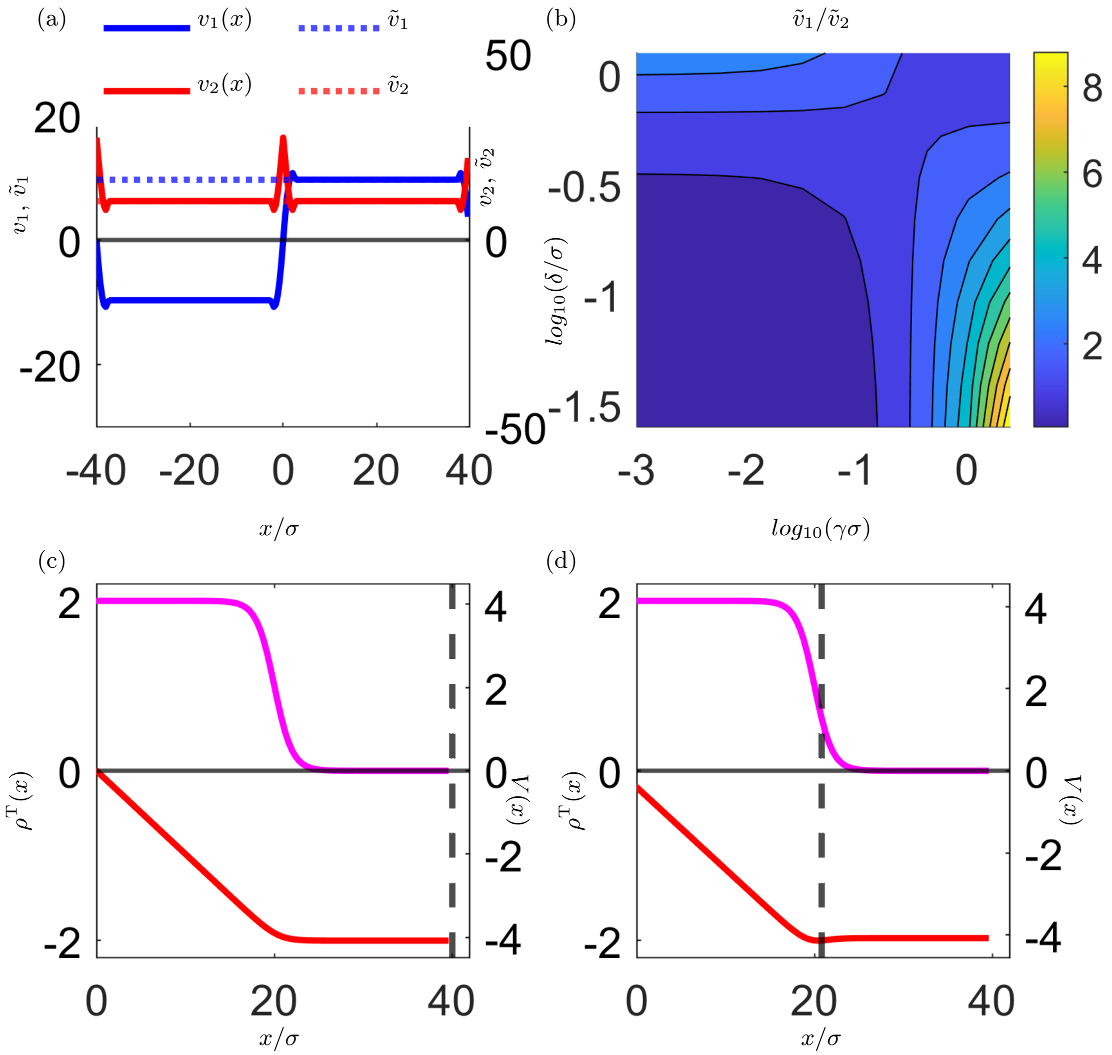

FIG. S5: (a) The functions $v_1(x;\gamma,\delta)$ and $v_2(x;\gamma,\delta)$ defined in the Methods, and their approximated amplitudes $\tilde{v}_1(\gamma,\delta)$ and $\tilde{v}_2(\gamma,\delta)$; the dependence on γ and δ was omitted in the legend for the sake of visibility. (b) The ratio of the amplitudes of v_1 and v_2, $\tilde{v}_1/\tilde{v}_2$ as a function of the control parameters γ and δ. As explained in Sec. II C, $\tilde{v}_1/\tilde{v}_2$ determines the steepness σ/Δ [Fig. 4(d) of the main text]. (c-d) Representative hyperbolic-tangent profile of $\rho^{\mathrm{T}}(x)$ (magenta line) and respective effective potential $V(x)$ (red line) as defined in Eq. (S31) for (c) $\tilde{v}_1 = 0.1$ and $\tilde{v}_2 = 0$, and (d) $\tilde{v}_1 = 0.1$, $\tilde{v}_2 = 0.2$. Dashed black lines indicate the position of the minimum of $V(x)$. Increasing $\tilde{v}_2$, the position of the minimum of $V(x)$ moves towards regions where ρ^{T} assumes a larger value.

III. PATTERN DESIGN BEYOND SIMPLE CONTAINMENT

In the main text we discussed how our continuum framework, beyond describing the specific shepherding task illustrated in Fig. 1 of the main text could also be used to model pattern formation in more general decision-making

systems (see Fig. 5 of the main text). To this end we propose to vary the spatial profile of the function $v_1(x)$ that multiplies the product of ρ^{T} and ρ^{H} in Eq. (3) of the main text. In other words, we consider this function as an *adjustable* control input whose shape can be varied to generate a desired pattern. Below we first discuss the explicit choices for $v_1(x)$ underlying the continuum results in Fig. 5 of the main text. Second, we describe strategies to translate these continuum rules to the agent-based level of description, yielding the microscopic results in Fig. 5 of the main text.

A. Continuum: choices for $v_1(x)$

In all of the four cases considered in Fig. 5 of the main text, the function $v_1(x)$ is a square wave with different periods or phase shifts, while $v_2(x)$ is set to a positive constant, i.e., $v_2(x) = \tilde{v}_2 > 0$. With these choices, the herder dynamics is given by

$$\partial_t \rho^{\mathrm{H}} = \nabla \cdot [D^{\mathrm{H}}(\rho^{\mathrm{H}})\nabla\rho^{\mathrm{H}} - v_1(x)\rho^{\mathrm{H}}\rho^{\mathrm{T}} - \tilde{v}_2\rho^{\mathrm{H}}\nabla\rho^{\mathrm{T}}] \tag{S32}$$

The target dynamics remains unchanged, its evolution equation is given by (see Eq. (4) in the main text)

$$\partial_t \rho^{\mathrm{T}} = \nabla \cdot \left[D^T(\rho^{\mathrm{T}})\nabla\rho^{\mathrm{T}} + \tilde{k}^T\rho^{\mathrm{T}}\nabla\rho^{\mathrm{H}}\right]. \tag{S33}$$

The concrete choices for $v_1(x)$ in Eq. (S32) corresponding to the four cases in Fig. 5 (main text) are as follows:

- **Containment**
This case corresponds to the shepherding task discussed in the main part: herders need to collect the targets in a goal region around $x = 0$. In the present study we have *derived* $v_1(x)$ from our agent-based model, yielding the expressions given in Eq. (15) of the Methods. As a simplified ansatz, we can use

$$v_1(x) = \tilde{v}_1 \mathrm{sign}(x) = \tilde{v}_1 \mathrm{sign}\left(\sin\left(\frac{2\pi x}{L}\right)\right) \tag{S34}$$

Here, the appearance of the sin-function in the argument could be replaced by a simpler function that is uneven in x; e.g., just by x itself. The ansatz (S34) is particularly suitable for comparison with the case of static patterns discussed below. Irrespective of this detail, Eq. (S34) generates currents of ρ^{H} to the "right" (to the "left") if a non-zero $\rho^{\mathrm{T}}(x)$ is observed for $x > 0$ ($x < 0$), eventually generating an accumulation of the herders around $x = \pm L/2$. The herder-target repulsion then pushes the targets toward the goal region around $x = 0$.

- **Expulsion**
In another application, herders may want to push targets *away* from a predefined (potentially dangerous) region. This can be realized by reversing the rule given in Eq. (S34), such that

$$v_1(x) = -\tilde{v}_1 \mathrm{sign}\left(\sin\left(\frac{2\pi x}{L}\right)\right) \tag{S35}$$

This choice of $v_1(x)$ generates currents of ρ^{H} to the "right" (to the "left") if a non-zero $\rho^{\mathrm{T}}(x)$ is observed for $x < 0$ ($x > 0$), eventually leading to the accumulation of the herders around $x = 0$. Targets are then expelled towards the boundary.

Notice that, given the periodicity of the domain, containment around x^* is equivalent to the expulsion from $x^* \pm L/2$: indeed the v_1-functions for containment [Eq. (S34)] and expulsion [Eq. (S35)] are the same but translated by $L/2$. This means that the two tasks are mathematically equivalent on this level of description.

The two cases discussed so far show that a change of sign of the function $v_1(x)$ around the position x^* produces containment of targets around x^* if $(x - x^*)v_1(x) \geq 0$ (for all $x \neq x^*$), or expulsion from the region around x^* if $(x - x^*)v_1(x) \leq 0$ (for all $x \neq x^*$). This suggests that more sophisticated patterns can be generated by including more zeros into $v_1(x)$. An example is given in the third case considered in Fig. 5 (main text):

- **Static patterns**
We consider a system where herders want to collect targets at several regions centered at positions x_j^* in the domain, where j is a positive integer going from 1 to the number of regions where to collect the targets. As a consequence, the targets will be expelled by the other regions of the domain. In a one-dimensional setup, the

desired resulting state would be characterized by a stripe pattern. As an example, we choose $x^*_{1,2} = \pm L/3$ and $x^*_3 = 0$ as the positions around which the targets shall be accumulated. This can be realized with

$$v_1(x) = \tilde{v}_1 \text{sign}\left(\sin\left(\frac{6\pi x}{L}\right)\right) \tag{S36}$$

whose zeros $x^*_{1,2,3}$ satisfy the condition $(x - x^*_j)v_1(x) \geq 0$ for x in a neighborhood of x^*_j $(j = 1, 2, 3)$.

- **Traveling patterns**
  Finally, it is interesting to consider the simplest choice for the function $v_1(x)$, namely

$$v_1(x) = \tilde{v}_1 = const \tag{S37}$$

  Indeed, as shown in Fig. 5 in the main text, a suitable choice for the constant $\tilde{v}_1$ produces a traveling pattern, where the herders persist to push the targets across the domain. A corresponding linear stability analysis predicting the occurrence of time-dependent motion is given in Sec. III C.

  Intuitively, we can understand the travelling patterns as follows: If $\tilde{v}_1 > 0$ (and $v^0_2 > 0$), the herders will move towards the "right" as long as they observe a non-zero $\rho^{\mathrm{T}}(x)$. This will eventually lead to the accumulation of the herders at the "right" of the targets. However, this cannot be a steady state since the coupling $-\tilde{v}_2\rho^{\mathrm{H}}\nabla\rho^{\mathrm{T}}$ pushes the herders towards the "left" where the targets are concentrated. This, in turn, will push the target even more towards the "left" as they try to escape the herders [due to the coupling $\tilde{k}^{\mathrm{T}}\rho^{\mathrm{T}}\nabla\rho^{\mathrm{H}}$ in the target dynamics, Eq. (S33)]. As a result, we observe traveling waves to the "left".

B. Agent dynamics: decison-making rules

We now show how to "translate" the design rules represented by the different functions $v_1(x)$ discussed in the preceding section towards the microscopic level of description. To this end we properly design the feedback control input $\mathbf{u}_i$ and combine these expressions with the agent-based Langevin equations given in Eqs. (7-8) of the Methods. To design the control input, we use the same general strategy as in the shepherding case considered in the main text. In particular, the herder's decision-making involves two key elements, namely (i) a selection rule for targets (whose sensitivity is tuned by γ) yielding $\mathbf{T}^*_i$, and (ii) a planning of the trajectory (controlled by δ). The recipes described below are the basis of the agent-based numerical results shown in Fig. 5 of the main text. All other parameters of the simulations are the same as those used for the simulations used to produce in Fig. 3 of the main text.

- **Containment**
  This is the same task analyzed in the main text (see Fig. 3) with the difference that we here employ a rectangular geometry for the goal region, different from the geometry used in the main text (see Sec. I D for the discussion on this point). Specifically, we use the following control input for the herders

$$\mathbf{u}_i = -\left(\mathbf{H}_i - \left(\mathbf{T}^{*,\mathrm{cont}}_i + \delta\,\text{sign}(\mathbf{T}^{*,\mathrm{cont}}_{i,x})\right)\right) \tag{S38}$$

  where

$$\mathbf{T}^{*,\mathrm{cont}}_i = \frac{\sum_{a\in N_{i,\xi}} e^{\gamma(|\mathbf{T}_{a,x}|-|\mathbf{H}_{i,x}|)}\mathbf{T}_a}{\sum_{a\in N_{i,\xi}} e^{\gamma(|\mathbf{T}_{a,x}|-|\mathbf{H}_{i,x}|)}}, \tag{S39}$$

  These rules eventually generate an accumulation of the herders around $x = \pm L/2$, pushing the targets into the region around $x = 0$.

- **Expulsion**
  As discussed above at the continuum level (see Sec. III A), containment around a given position x^* is equivalent to the expulsion from $x^* \pm L/2$ (which are the points with largest distance from x^*) due to the periodicity of the domain. As a consequence, to generate expulsion from $x = 0$ we design the selection rule so as to select the target with the largest distance from $x^* = L/2$ (which coincides with $x^* = -L/2$). This yields

$$\mathbf{T}^{*,\exp}_i(\gamma) = \frac{\sum_{a\in N_{i,\xi}} e^{\gamma((L/2-|\mathbf{T}_{a,x}|)-(L/2-|\mathbf{H}_{i,x}|))}\mathbf{T}_a}{\sum_{a\in N_{i,\xi}} e^{\gamma((L/2-|\mathbf{T}_{a,x}|)-(L/2-|\mathbf{H}_{i,x}|))}} = \frac{\sum_{a\in N_{i,\xi}} e^{-\gamma(|\mathbf{T}_{a,x}|-|\mathbf{H}_{i,x}|)}\mathbf{T}_a}{\sum_{a\in N_{i,\xi}} e^{-\gamma(|\mathbf{T}_{a,x}|-|\mathbf{H}_{i,x}|)}} = \mathbf{T}^{*,\mathrm{cont}}_i(-\gamma) \tag{S40}$$

The third and fourth member of these equations show that selection of target the largest distance from $x^* = L/2$ is equivalent to the target selection rule Eq. (S38) we used for containment, yet with negative γ. Indeed, the choice $\gamma < 0$ implies selection of the target with the smallest (rather than the largest) distance from the origin. Regarding the trajectory planning, following the same reasoning, we want the herders to place themselves *between* the selected target and the origin. Therefore, we can again use the same formulation as in the containment case [Eq. (S38)], but now the herders place themselves with a shift $-\delta\,\mathrm{sign}(\mathbf{T}^*_{i,x})$. The control input then becomes

$$\mathbf{u}_i = -\left(\mathbf{H}_i - (\mathbf{T}^*_i - \delta\,\mathrm{sign}\mathbf{T}^{*,\mathrm{cont}}_{i,x}(-\gamma)\right) \tag{S41}$$

with $0 < \delta < \lambda$, and $1/\xi \ll \gamma$.

- **Static patterns**
To create a static stripe-like pattern, we follow the strategy described on the continuum level where $v_1(x)$ was chosen as a square wave whose reflection points correspond to the centers of containment regions. In other words, the task of creating a stripe patterns is divided into several containment tasks, each involving one "accumulation point" x^*. This motivates to formulate the selection rule as follows: If one herder, say herder i, observes a target a in its sensing region, it first identifies the accumulation points x^* to which this herder and the target are the closest (among the set of accumulation points). We refer to these two special accumulation points as x^*_i and x^*_a, respectively. The selection rule can then be written as

$$\mathbf{T}^*_i = \frac{\sum_{a\in N_{i,\xi}} e^{\gamma(|\mathbf{T}_{a,x}-x^*_a|-|\mathbf{H}_{i,x}-x^*_i|)}\mathbf{T}_a}{\sum_{a\in N_{i,\xi}} e^{\gamma(|\mathbf{T}_{a,x}-x^*_a|-|\mathbf{H}_{i,x}-x^*_i|)}} \tag{S42}$$

For the trajectory planning, we accordingly require the herder to place itself at the back of the target with respect to x^*_a. We note that x^*_a can vary during the motion of all agents (contrary to the fixed goal regions considered in the other cases). Therefore, we write the control input as

$$\mathbf{u}_i = -\left(\mathbf{H}_i - \mathbf{H}^*_i(\gamma,\delta)\right) \tag{S43}$$

where $\mathbf{H}^*_i$ is the desired position for herder i defined as

$$\mathbf{H}^*_i(\gamma,\delta) = \frac{\sum_{a\in N_{i,\xi}} e^{\gamma(|\mathbf{T}_{a,x}-x^*_a|-|\mathbf{H}_{i,x}-x^*_i|)}(\mathbf{T}_a + \delta\mathrm{sign}(\mathbf{T}_{a,x}-x^*_a))}{\sum_{a\in N_{i,\xi}} e^{\gamma(|\mathbf{T}_{a,x}-x^*_a|-|\mathbf{H}_{i,x}-x^*_i|)}} \tag{S44}$$

Notice that this strategy requires the herders to know the positions of the multiple accumulation points x^*.

- **Travelling patterns**
As discussed in the continuum part, numerical simulations of the field equations with $v_1(x) = \tilde{v}_1$ (as well as the corresponding linear stability analysis) reveal for suitable choices of the constants $\tilde{v}_1$ and $\tilde{v}_2$ a travelling pattern. Specifically, for $\tilde{v}_1 > 0$, the coupling $\tilde{v}_1\rho^{\mathrm{T}}\rho^{\mathrm{H}}$ generates a persistent travelling wave moving to the "left". On the agent-based level, we can create such a situation with a selection rule that favors the target with the largest value of the x coordinate in the sensing region of herder i

$$\mathbf{T}^{*,\mathrm{trav\ patt}}_i = \frac{\sum_{a\in N_{i,\xi}} e^{\gamma(\mathbf{T}_{a,x}-\mathbf{H}_{i,x})}\mathbf{T}_a}{\sum_{a\in N_{i,\xi}} e^{\gamma(\mathbf{T}_{a,x}-\mathbf{H}_{i,x})}}, \tag{S45}$$

We combine this rule with the requirement that a herder always places itself to the "right" of the target, in the $x > 0$ direction. This is achieved by writing the distributed control input for herder i as

$$\mathbf{u}_i = -\left(\mathbf{H}_i - (\mathbf{T}^{*,\mathrm{trav\ patt}}_i + \delta)\right) \tag{S46}$$

C. Linear stability analysis for the case of constant coefficients $\tilde{v}_1$ and $\tilde{v}_2$

Putting the functions $v_1(x)$ and $v_2(x)$ in the full herder dynamics to constants, we arrive at the coupled PDEs

$$\partial_t\rho^{\mathrm{T}} = \nabla\cdot\left[D^T(\rho^{\mathrm{T}})\nabla\rho^{\mathrm{T}} + \tilde{k}^T\rho^{\mathrm{T}}\nabla\rho^{\mathrm{H}}\right] \tag{S47}$$

$$\partial_t\rho^{\mathrm{H}} = \nabla\cdot\left[D^H(\rho^{\mathrm{H}})\nabla\rho^{\mathrm{H}} - \tilde{v}_1\rho^{\mathrm{H}}\rho^{\mathrm{T}} - \tilde{v}_2\rho^{\mathrm{H}}\nabla\rho^{\mathrm{T}}\right] \tag{S48}$$

We first note that a trivial steady state solution for the above equations is given by $\rho^{\mathrm{T}}(x) = \rho_0^{\mathrm{T}}$, $\rho^{\mathrm{H}}(x) = \rho_0^{\mathrm{H}}$, corresponding to a homogeneous steady state. This is in stark contrast to the case of a space-dependent function $v_1(x)$ that does not allow for such a steady state, as discussed in the main text (see Eq. (6)).

We now perform a linear stability analysis (LSA) around the homogeneous solution, following the procedure outlined in Sec. II B (where we considered the system without decision-making). Introducing small fluctuations $\delta\rho^{\mathrm{A}}(x,t)$ with $\mathrm{A} = \mathrm{H,T}$ [see Eq. (S23)] and linearizing Eqs. (S48) we obtain

$$\partial_t \delta\rho^{\mathrm{T}}(x,t) = D^{\mathrm{T}}(\rho_0^{\mathrm{T}})\nabla^2\delta\rho^{\mathrm{T}}(x,t) + \rho_0^{\mathrm{T}}\tilde{k}^{\mathrm{T}}\nabla^2\delta\rho^{\mathrm{H}}(x,t) \tag{S49}$$

$$\partial_t \delta\rho^{\mathrm{H}}(x,t) = D^{\mathrm{H}}(\rho_0^{\mathrm{H}})\nabla^2\delta\rho^{\mathrm{H}}(x,t) - \tilde{v}_2\rho_0^{\mathrm{H}}\nabla^2\delta\rho^{\mathrm{T}}(x,t) - \tilde{v}_1\left(\rho_0^{\mathrm{T}}\nabla\delta\rho^{\mathrm{H}}(x,t) + \rho_0^{\mathrm{H}}\nabla\delta\rho^{\mathrm{T}}(x,t)\right) \tag{S50}$$

Next, we expand the fluctuations in a Fourier series [see Eq. (S26)]. Insertion into Eqs. (S50) and (S49) brings us to the eigenvalue problem

$$-q\begin{bmatrix} qD^{\mathrm{T}}(\rho_0^{\mathrm{T}}) & q\tilde{k}^{\mathrm{T}}\rho_0^{\mathrm{T}} \\ -q\tilde{v}_2\rho_0^{\mathrm{H}} + i\tilde{v}_1\rho_0^{\mathrm{H}} & qD^{\mathrm{H}}(\rho_0^{\mathrm{H}}) + i\tilde{v}_1\rho_0^{\mathrm{T}} \end{bmatrix}\mathbf{v}(q) = \Omega(q)\mathbf{v}(q) \tag{S51}$$

where we note the different dependency on the wavenumber q as compared to the uncontrolled case [see Eq. (S27)]. A further difference is that the two matrix elements involving $\tilde{v}_1$ are complex (due to the product of densities appearing in the related current). As a consequence, if $\tilde{v}_1 \neq 0$, the growth rate $\Omega(q)$ has always an imaginary part.

To decide about the (linear) stability of the homogeneous steady state, we have to consider the real part of the growth rate, $\mathrm{Re}(\Omega)(q)$. We have performed extensive numerical investigations for a range of the parameters $\tilde{v}_1$ and the strength of herder-target repulsion, $\tilde{k}^{\mathrm{T}}$. Results for three wavenumbers q and two values of $\tilde{v}_2$ (one positive, one negative) are presented in Fig. S6. In the yellow regions, $\mathrm{Re}(\Omega)(q) > 0$ which, together with the nonzero imaginary parts, signals a temporal (oscillatory) instability.

We first consider the case $\tilde{v}_2 > 0$ (top row of Fig. S6), where herders are attracted to the targets (as assumed throughout the rest of this paper). The results show that, for sufficiently strong herder-target repulsion $\tilde{k}^{\mathrm{T}}$ (such as the value considered in the main text, indicated by an orange star), an increase of $|\tilde{v}_1|$ from zero can produce a temporal instability. An exception is the special case $\tilde{v}_1 = 0$ where, consistent with our results in Fig. S4, no temporal instability occurs. We further observe from Fig. S6(a-c) that the (yellow) regimes indicating positive growth rates shrink with increasing wavenumber (and completely vanish for large enough q). This can be understood from the fact that the instability is driven by $\tilde{v}_1$ (providing a term linear in q in Eq. (S51) which competes with all of the other terms that provide a q^2-contribution in Fourier space (which, as discussed in Sec. II B, would otherwise make the homogeneous state stable).

Turning to the case $\tilde{v}_2 < 0$ (where herders are, on average, repelled by targets), the situation is different, as revealed by the diagrams in the bottom row of Fig. S6). Here, it is seen that the system is temporarily unstable even for $\tilde{v}_1 = 0$. This already indicates that for $\tilde{v}_2 < 0$, the instability is driven by the repulsion between targets and herders (modulated by $\tilde{k}^{\mathrm{T}}$), represented by a term proportional to q^2 in Eq. (S51). Accordingly, the larger q, the larger the region of the parameters where the homogeneous steady state is unstable.

Finally, we recall that travelling patterns are one of the key effects observed in nonreciprocal systems, e.g., in the Cahn-Hillard equations for phase-separating systems if the nonreciprocity in the couplings between the fields is sufficiently pronounced [9, 13, 16]. Here we obtain such an oscillatory instability even the case that there is no source of phase separation (such as attraction between particles of one species).

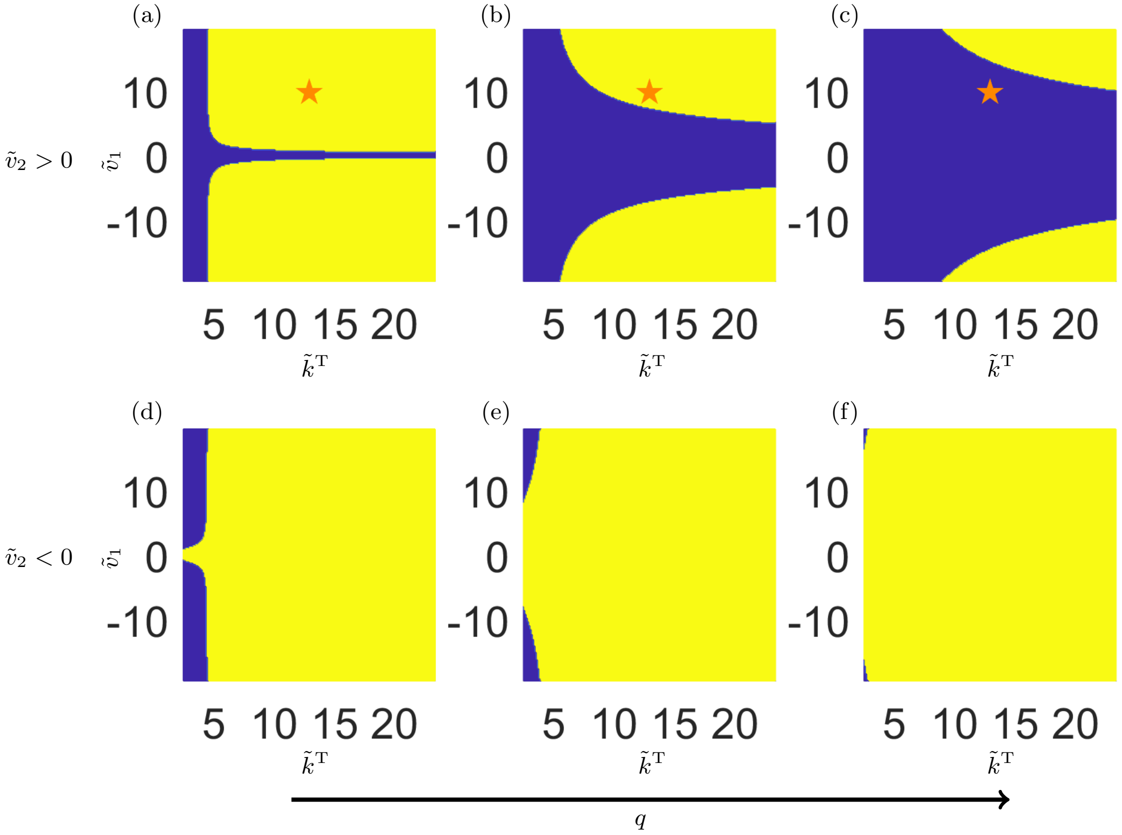

FIG. S6: LSA of the field equations S48 and S47 with respect to a homogeneous steady state. Yellow regions indicate where one of growth rates obtained from Eq. S51 has a positive real part. We present results for both $\tilde{v}_2 > 0$ (a-c) and $\tilde{v}_2 < 0$ (d-f), and for different values of the wavenumber q, increased from left to right with values $q = \frac{2\sigma\pi}{L}[1, 10, 20]$. In all cases, the corresponding imaginary part is non-zero. (a-c) $\tilde{v}_2 > 0$: this is the case discussed in the main text, where herders are attracted to the targets on the field level. The results show that we cannot achieve a positive real part of the growth rates for $\tilde{v}_1 = 0$, consistently with Fig. S4. (d-f) $\tilde{v}_2 < 0$: in this case herders and targets both repel each other. The regions of instability increase with q, differently from the case $\tilde{v}_2 > 0$. The parameter values used in (a-c) are the same as those used in Fig. 5(l) of the main text (see Methods), except for the two varied parameters $\tilde{k}^{\mathrm{T}}$ and $\tilde{v}_1$. The precise values related to Fig. 5(l) of the main text correspond to the the orange star. To reproduce panels (d-f), the same values are used, except for k^{H} whose sign needs to be inverted.

IV. LIST OF SUPPLEMENTARY VIDEOS

1. Supplementary Information Video 1: numerical simulations of the agent-based equations (7-8) of the main text, showing the emergence of containment starting from a homogeneous configuration. The parameters γ and δ are set to the largest considered values, namely $\gamma = 10/\sigma$, $\delta = \lambda/2$. Blue diamonds represent the herders, magenta dots represent the targets.

2. Supplementary Information Video 2: Numerical simulations of the field equations (3-4) of the main text showing the emergence of containment at the continuum level starting from a homogeneous state. The parameters γ and δ are set to the largest considered values, namely $\gamma = 2.5/\sigma$, $\delta = \lambda/2$. The blue and magenta lines respectively represent ρ^{H} and ρ^{T}.

3. Supplementary Information Video 3: numerical simulations of the field equations Eq. (S47) and Eq. (S48) showing the emergence of traveling patterns from a perturbed homogeneous state. The numerical values of the parameters are presented in the Methods. The blue and magenta lines represent ρ^{H} and ρ^{T}, respectively.

4. Supplementary Information Video 4: agent-based numerical simulations showing the emergence of traveling patterns (see Section III B). Blue diamonds represent the herders, magenta dots represent the targets.

[1] A. Lama and M. di Bernardo, Shepherding and herdability in complex multiagent systems, Physical Review Research **6**, L032012 (2024).

[2] P. Nalepka, R. W. Kallen, A. Chemero, E. Saltzman, and M. J. Richardson, Herd those sheep: Emergent multiagent coordination and behavioral-mode switching, Psychological science **28**, 630 (2017).

[3] S. Zhang, X. Lei, M. Duan, X. Peng, and J. Pan, A distributed outmost push approach for multirobot herding, IEEE Transactions on Robotics **40**, 1706 (2024).

[4] F. Auletta, D. Fiore, M. J. Richardson, and M. di Bernardo, Herding stochastic autonomous agents via local control rules and online target selection strategies, Autonomous Robots **46**, 469 (2022).

[5] G. Ariel and A. Ayali, Locust collective motion and its modeling, PLOS computational Biology **11**, e1004522 (2015).

[6] A. Cavagna, A. Culla, X. Feng, I. Giardina, T. S. Grigera, W. Kion-Crosby, S. Melillo, G. Pisegna, L. Postiglione, and P. Villegas, Marginal speed confinement resolves the conflict between correlation and control in collective behaviour, Nature Communications **13**, 2315 (2022).

[7] G. Sartoretti, M.-O. Hongler, M. E. de Oliveira, and F. Mondada, Decentralized self-selection of swarm trajectories: from dynamical systems theory to robotic implementation, Swarm Intelligence **8**, 329 (2014).

[8] G. Vásárhelyi, C. Virágh, G. Somorjai, T. Nepusz, A. E. Eiben, and T. Vicsek, Optimized flocking of autonomous drones in confined environments, Science Robotics **3**, eaat3536 (2018).

[9] Z. You, A. Baskaran, and M. C. Marchetti, Nonreciprocity as a generic route to traveling states, Proceedings of the National Academy of Sciences **117**, 19767 (2020).

[10] N. Long, K. Sammut, D. Sgarioto, M. Garratt, and H. Abbass, A comprehensive review of shepherding as a bio-inspired swarm-robotics guidance approach], IEEE Transactions on Emerging Topics in Computational Intelligence **4**, 523 (2020).

[11] S. Van Havermaet, P. Simoens, T. Landgraf, and Y. Khaluf, Steering herds away from dangers in dynamic environments, Royal Society Open Science **10**, 230015 (2023).

[12] M. Schmidt and H. Löwen, Freezing between two and three dimensions, Physical review letters **76**, 4552 (1996).

[13] S. Saha, J. Agudo-Canalejo, and R. Golestanian, Scalar active mixtures: The nonreciprocal cahn-hilliard model, Physical Review X **10**, 041009 (2020).

[14] F. Brauns and M. C. Marchetti, Nonreciprocal pattern formation of conserved fields, Physical Review X **14**, 021014 (2024).

[15] R. Zwanzig, *Nonequilibrium statistical mechanics* (Oxford university press, 2001).

[16] M. Fruchart, R. Hanai, P. B. Littlewood, and V. Vitelli, Non-reciprocal phase transitions, Nature **592**, 363 (2021).
