# Supplementary material for: Nonreciprocal field theory for decision-making in multi-agent control systems
Source: Nat Commun. 2025 Sep 26;16:8450. doi: 10.1038/s41467-025-63071-4 (PMC12474890; doi:10.1038/s41467-025-63071-4)
Supplement: Supplementary file 2 — Description of Additional Supplementary Files [file 41467_2025_63071_MOESM2_ESM.pdf]

Description of Additional Supplementary Files

Supplementary Video 1: Numerical simulations of the agent-based equations (7-8) of the main text, showing the emergence of containment starting from a homogeneous configuration. The parameters γ and δ are set to the largest considered values, namely $\gamma = 10/\sigma$, $\delta = \lambda/2$. Blue diamonds represent the herders, magenta dots represent the targets.

Supplementary Video 2: Numerical simulations of the field equations (3-4) of the main text showing the emergence of containment at the continuum level starting from a homogeneous state. The parameters γ and δ are set to the largest considered values, namely $\gamma = 2.5/\sigma$, $\delta = \lambda/2$. The blue and magenta lines respectively represent ρ H and ρ T .

Supplementary Video 3: Numerical simulations of the field equations showing the emergence of traveling patterns from a perturbed homogeneous state. The numerical values of the parameters are presented in the Methods. The blue and magenta lines represent ρ H and ρ T , respectively.

Supplementary Video 4: Agent-based numerical simulations showing the emergence of traveling patterns. Blue diamonds represent the herders, magenta dots represent the targets.
